# Expression of LDL receptor-related proteins (LRPs) in common solid malignancies correlates with patient survival

**DOI:** 10.1371/journal.pone.0186649

**Published:** 2017-10-31

**Authors:** Steven L. Gonias, Nicki Karimi-Mostowfi, Sarah S. Murray, Elisabetta Mantuano, Andrew S. Gilder

**Affiliations:** 1 Department of Pathology, University of California San Diego, La Jolla, California, United States of America; 2 The Department of Experimental Medicine, Sapienza University of Rome, Rome, Italy; Duke University School of Medicine, UNITED STATES

## Abstract

LDL receptor-related proteins (LRPs) are transmembrane receptors involved in endocytosis, cell-signaling, and trafficking of other cellular proteins. Considerable work has focused on LRPs in the fields of vascular biology and neurobiology. How these receptors affect cancer progression in humans remains largely unknown. Herein, we mined provisional databases in The Cancer Genome Atlas (TCGA) to compare expression of thirteen LRPs in ten common solid malignancies in patients. Our first goal was to determine the abundance of LRP mRNAs in each type of cancer. Our second goal was to determine whether expression of LRPs is associated with improved or worsened patient survival. In total, data from 4,629 patients were mined. In nine of ten cancers studied, the most abundantly expressed LRP was *LRP1*; however, a correlation between *LRP1* mRNA expression and patient survival was observed only in bladder urothelial carcinoma. In this malignancy, high levels of *LRP1* mRNA were associated with worsened patient survival. High levels of LDL receptor (*LDLR*) mRNA were associated with decreased patient survival in pancreatic adenocarcinoma. High levels of *LRP10* mRNA were associated with decreased patient survival in hepatocellular carcinoma, lung adenocarcinoma, and pancreatic adenocarcinoma. *LRP2* was the only LRP for which high levels of mRNA expression correlated with improved patient survival. This correlation was observed in renal clear cell carcinoma. Insights into LRP gene expression in human cancers and their effects on patient survival should guide future research.

## Introduction

The LDL Receptor (*LDLR*) gene family includes single-pass, type 1 transmembrane proteins that share common structural motifs, including EGF-like repeats, cysteine-rich complement-like repeats, and sequential YWTD-containing repeats that are organized into β-propeller structures [1–5]. Although members of this gene family, which are typically called LDL Receptor-related Proteins (LRPs), may be more closely or distantly related to the LDLR, for the purposes of this study, we consider thirteen LRPs, including the LDLR, LRP1/CD91, LRP1b, LRP2/megalin, LRP3, LRP4/MEGF7, LRP5, LRP6, LRP8/apolipoprotein E receptor 2, LRP10, which also has been referred to as LRP9 [6], LRP11/SorLA, LRP12/ST7, and the VLDL receptor (VLDLR). At the cellular level, LRPs function in endocytosis, cargo transport, and cell-signaling, and regulate the subcellular localization of other proteins [3–5, 7–9].

Considerable work has focused on the function of LRPs in vascular biology and neurobiology. LRPs also have been studied in cancer; however, how these receptors affect cancer progression in human patients remains largely unknown. Published work on LRP1 provides an example. Preclinical studies in cell culture and mouse model systems have shown that LRP1 may decrease the aggressiveness of cancer cells by down-regulating the cell-surface abundance of urokinase-type plasminogen activator receptor (uPAR), by internalizing metalloproteinases (MMPs), and by activating cell-signaling pathways that counteract β-catenin-signaling [10–13]. On the other hand, LRP1 may promote cancer progression by serving as a receptor for the growth factor, midkine, by signaling through ERK1/2 to induce expression of MMPs, and by facilitating survival of micro-metastases [10, 14–16].

The Cancer Genome Atlas (TCGA) is an open access resource that provides transcriptome profiling databases for diverse solid malignancies in human patients [17]. TCGA mRNA expression data collected using RNA next-generation sequencing (RNA-Seq) may be mined to compare expression of genes within a large dynamic range [18]. The goal of the present study was to determine whether expression of LRPs within tumor tissue has predictive value with regard to prognosis and/or outcome in human cancers. We examined a wide scope of common solid malignancies including bladder urothelial carcinoma, breast invasive carcinoma, colorectal adenocarcinoma, glioblastoma, renal clear cell carcinoma, hepatocellular carcinoma, lung adenocarcinoma, pancreatic adenocarcinoma, prostate adenocarcinoma, and cutaneous melanoma. Our analysis identified correlations between *LRP1* and *LRP2* mRNA expression levels and patient survival, but only in bladder urothelial carcinoma and renal clear cell carcinoma, respectively. Increased *LRP10* mRNA expression was associated with decreased patient survival in three different malignancies: hepatocellular carcinoma, lung adenocarcinoma, and pancreatic adenocarcinoma. Increased *LDLR* mRNA expression was associated with decreased patient survival in pancreatic adenocarcinoma. These results provide justification for further research to elucidate the function of LRPs in cancer.

## Results

### Variation in gene expression in specimens of a single type of cancer and amongst different cancers

To study expression of LRPs in solid malignancies in humans and determine whether expression of LRPs is associated with altered patient survival, we mined provisional TCGA datasets. The types of cancer analyzed and the number of patients in each provisional dataset at the time of analysis are shown in Table 1. Our first objective was to compare absolute expression of different LRP mRNAs within each solid malignancy using RNA-Seq data, quantified and normalized using the RSEM algorithm [19]. The same data were transformed to examine expression of individual LRPs in different cancers. In this study, we did not compare expression of LRPs in paired samples from non-malignant tissue adjacent to tumors or normal tissue.

**Table 1:** Solid malignancies analyzed using cBioPortal (TCGA, provisional).

Cancer	Number of Patients
Bladder Urothelial Carcinoma	408
Breast Invasive Carcinoma	1100
Colorectal Adenocarcinoma	382
Glioblastoma	166
Renal Clear Cell Carcinoma	534
Hepatocellular Carcinoma	373
Lung Adenocarcinoma	517
Pancreatic Adenocarcinoma	179
Prostate Adenocarcinoma	498
Cutaneous Melanoma	472

To provide a framework for interpreting variability in LRP gene expression amongst specimens of a single type of cancer and in different cancers, we mined the ten TCGA datasets to identify genes that demonstrate the least variability. First, we examined individual cancer datasets. For each gene, the coefficient of variation (CV = SD/mean) in mRNA expression was determined. The genes were then ranked, beginning with the lowest CV, which indicates the lowest degree of variability in gene expression from tumor sample to tumor sample. When the absolute expression level of a gene was below the median expression level for all genes in a dataset, in three or more of the ten cancers studied, that gene was omitted from the analysis. Three genes emerged: *TARDP* (Transcription of RNA activating protein/TAR DNA binding protein); *HNRNPK* (Heterogeneous Nuclear Ribonucleoprotein K); and *WDR33* (WD Repeat Domain 33) as having the lowest CVs across the spectrum of cancers (Table 2). For *TARDP*, the CVs ranged from 0.118–0.202 (mean of 0.172). For *HNRNPK* and *WDR33*, the CVs varied from 0.141–0.215 (mean of 0.182) and from 0.136–0.252 (mean of 0.210), respectively. For comparison, the mean CVs for the frequently studied gene expression standards, *GAPDH* (glyceraldehyde 3-phosphate dehydrogenase) and *ACTB* (β-actin), were 0.587 and 0.369, respectively.

**Table 2:** Identification of three novel gene standards for assessing variability in gene expression in human cancer.

***TARDBP***				
**Tumor**	**Mean**	**ST DEV**	**CV**	**CV Rank**
Urothelial Carcinoma	3828.70	670.66	0.175	1
Breast Invasive Carcinoma	3611.89	546.72	0.151	1
Colorectal Adenocarcinoma	3532.25	615.47	0.174	11
Hepatocellular Carcinoma	3011.73	557.66	0.185	1
Renal Clear Cell Carcinoma	2806.08	520.45	0.185	21
Lung Adenocarcinoma	3165.96	618.77	0.195	2
Cutaneous Melanoma	3258.84	659.13	0.202	1
Pancreatic Adenocarcinoma	3085.40	365.37	0.118	1
Prostate Adenocarcinoma	3398.49	446.96	0.132	2
Glioblastoma	3049.98	607.92	0.199	86
Mean CV = 0.172				
Mean expression (±SD) in ten cancers: 3274 ± 312, CV = 0.095				
***HNRNPK***				
**Tumor**	**Mean**	**ST DEV**	**CV**	**CV Rank**
Urothelial Carcinoma	11828.69	2549.08	0.215	4
Breast Invasive Carcinoma	13805.55	2454.08	0.178	2
Colorectal Adenocarcinoma	13099.82	1852.45	0.141	1
Hepatocellular Carcinoma	10051.10	2146.73	0.214	3
Renal Clear Cell Carcinoma	11289.79	1683.60	0.149	1
Lung Adenocarcinoma	11029.18	1969.11	0.179	1
Cutaneous Melanoma	11583.02	2483.32	0.214	3
Pancreatic Adenocarcinoma	10448.15	1493.86	0.143	6
Prostate Adenocarcinoma	12075.11	2317.51	0.192	201
Glioblastoma	10164.33	2021.06	0.199	80
Mean CV = 0.182				
Mean expression (±SD) in ten cancers: 11537 ± 1227, CV = 0.106				
***WDR33***				
**Tumor**	**Mean**	**ST DEV**	**CV**	**CV Rank**
Urothelial Carcinoma	1388.32	296.64	0.214	3
Breast Invasive Carcinoma	1350.54	276.37	0.205	5
Colorectal Adenocarcinoma	1434.31	235.41	0.164	3
Hepatocellular Carcinoma	1345.56	307.63	0.229	6
Renal Clear Cell Carcinoma	1299.26	233.45	0.180	15
Lung Adenocarcinoma	1602.65	404.10	0.252	49
Cutaneous Melanoma	1401.42	298.26	0.213	2
Pancreatic Adenocarcinoma	1344.65	182.35	0.136	4
Prostate Adenocarcinoma	1246.94	201.57	0.162	39
Glioblastoma	1027.06	177.02	0.172	11
Mean CV = 0.210				
Mean expression (±SD) in ten cancers: 1343 ± 146, CV = 0.109				
***GAPDH***				
**Tumor**	**Mean**	**ST DEV**	**CV**	**CV Rank**
Urothelial Carcinoma	90712	49119	0.541	2790
Invasive Carcinoma	56875	42011	0.739	3907
Colorectal Adenocarcinoma	78335	33163	0.423	3179
Hepatocellular Carcinoma	72330	51516	0.712	2361
Renal Clear Cell Carcinoma	116430	64085	0.550	3316
Lung Adenocarcinoma	58154	38372	0.660	3692
Cutaneous Melanoma	122930	83104	0.676	3494
Pancreatic Adenocarcinoma	57592	40059	0.696	3752
Prostate Adenocarcinoma	35117	15528	0.442	2353
Glioblastoma	96437	41842	0.434	2905
Mean CV = 0.587				
Mean expression (±SD) in ten cancers: 78488 ± 28134, CV = 0.358				
***ACTB***				
**Tumor**	**Mean**	**ST DEV**	**CV**	**CV Rank**
Urothelial Carcinoma	120546	53994	0.448	1958
Breast Invasive Carcinoma	98226	40396	0.411	1558
Colorectal Adenocarcinoma	113527	29562	0.260	710
Hepatocellular Carcinoma	74583	32199	0.432	1006
Renal Clear Cell Carcinoma	85639	30297	0.354	1628
Lung Adenocarcinoma	121276	43140	0.356	1032
Cutaneous Melanoma	122446	56282	0.460	1834
Pancreatic Adenocarcinoma	120746	38580	0.320	1510
Prostate Adenocarcinoma	65830	24318	0.369	2627
Glioblastoma	135424	39077	0.289	1078
Mean CV + 0.369				
Mean expression (±SD) in ten cancers: 105824 ± 23406, CV = 0.221				

Next, we compared expression of *TARDP*, *HNRNPK*, *and WDR33* in different cancers. The mean levels of mRNA expression were averaged and SDs were determined (n = 10). The CVs were 0.095, 0.106, and 0.109 for *TARDP*, *HNRNPK*, and *WDR33*, respectively, reflecting variability in gene expression across different categories of cancer that was no greater than the variability observed in different specimens of the same malignancy. These results suggest that there are genes that are expressed in a fairly consistent manner by cancers of different origin and derivation.

### LRP expression in solid malignancies

**Fig 1:**
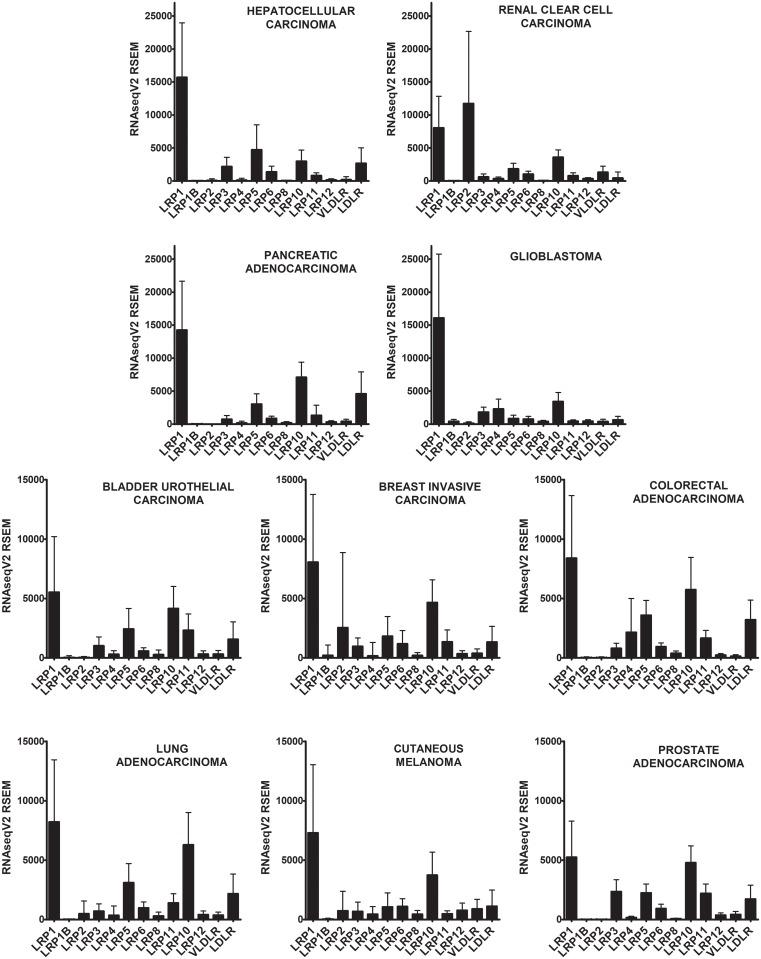
Relative abundance of LRPs in ten common solid malignancies. RNAseqV2 data for the indicated cancers are presented as the mean ± SD. Note that the scale for the y-axis is different in the top four graphs compared with the bottom six graphs.

Expression of thirteen LRPs was compared in ten solid malignancies (Fig 1). In nine of ten cancers, *LRP1* was the most abundantly expressed LRP at the mRNA level. The CVs for *LRP1* mRNA expression in individual cancers ranged from 0.52–0.79. In renal clear cell carcinoma, *LRP2* was the most abundantly expressed LRP. This result is interesting because LRP2/megalin was first characterized as a protein expressed by proximal tubule and glomerular epithelial cells in the kidney [20, 21]. Considerable variability in *LRP2* mRNA expression was observed in renal clear cell carcinoma (CV = 0.93). Other LRPs that were expressed at relatively high levels in a number of solid malignancies included *LRP10* and *LRP5*.

**Fig 2:**
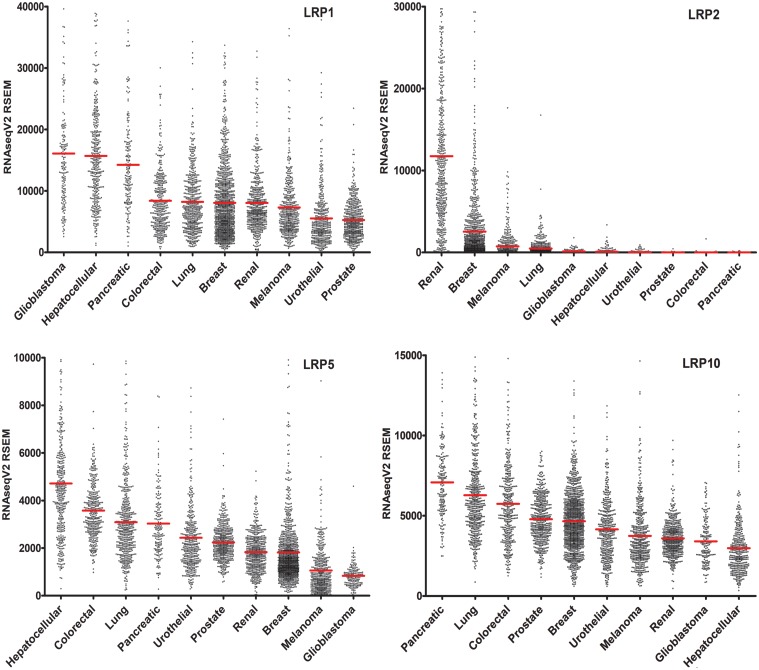
Vertical scatter plots comparing LRP expression in different cancers. Results are presented for *LRP1*, *LRP2*, *LRP5*, and *LRP10*. Each tumor specimen is represented by a data point. Red horizontal bars mark the mean expression level. To optimize data presentation, a small number of points demarcating tumors with “off-scale” high mRNA expression were omitted as follows: *LRP1*: glioblastoma (5), hepatocellular carcinoma (5), other tumors (3); *LRP2*: renal clear cell carcinoma (27), breast carcinoma (12); *LRP5*: breast carcinoma (8), hepatocellular carcinoma (7), urothelial carcinoma (5), other tumors (6); *LRP10*: lung adenocarcinoma (9); other tumors (3).

Next, we re-organized the mRNA expression data to compare the relative abundance of specific LRP mRNAs in different cancers. Fig 2 shows results for *LRP1*, *LRP2*, *LRP5* and *LRP10*. We selected these LRPs for presentation in Fig 2 because they were highly expressed in one or more solid malignancy. Similar comparisons for other LRPs may be derived from the data shown in Fig 1. *LRP1* mRNA was most abundant in glioblastoma, hepatocellular carcinoma, and pancreatic adenocarcinoma. In addition to renal clear cell carcinoma, *LRP2* mRNA was detected in breast invasive carcinoma at fairly high levels but with extremely high variability (CV = 2.43). *LRP5* mRNA was most abundantly expressed in hepatocellular, colorectal, lung, and pancreatic carcinoma. *LRP10* mRNA was most abundantly expressed in pancreatic, lung, and colorectal carcinoma. A pitfall that must be considered when analyzing the data presented in Fig 2 is the contribution of non-malignant cell mRNA to the expression results, which may be inconsistent in different malignancies. Non-malignant cells also may contribute differentially to the abundance of mRNAs for different LRPs, impacting the results presented in Fig 1.

### LRP expression in cancer and patient survival

Next, we mined available data to search for correlations between LRP mRNA expression and patient survival. For each cancer, we examined the LRPs that were most abundantly expressed. Cases for which survival data were available were stratified based on the expression level of the LRP of interest. Two comparisons were made. First, cases with mRNA expression levels above the median (top 50%) were compared with cases with mRNA expression levels below the median (bottom 50%). Second, cases with mRNA expression levels in the top quartile were compared with cases with LRP expression levels in the bottom quartile, discarding 50% of the cases from the analysis. Table 3 summarizes the results of the survival analyses performed.

**Table 3:** Patient survival analyses.

Tumor	LRP1	LRP2	LRP4	LRP5	LRP10	LDL-R
Urothelial	p<0.0005	X	X	NS	NS	X
Breast	NS	NS	X	NS	NS	X
Colorectal	NS	X	X	NS	NS	NS
Glioblastoma	NS	X	NS	X	NS	X
Renal Cell	NS	p<0.0001	X	X	NS	X
Hepatocellular	NS	X	X	NS	p<0.005	NS
Lung	NS	X	X	NS	p<0.05	NS
Pancreatic	NS	X	X	NS	p<0.05	p<0.01
Prostate	NS	X	X	X	NS	X
Melanoma	NS	NS	X	X	NS	X

P-values are shown for statistically significant differences in patient survival associated with having tumors in which expression of the indicated LRP is in the top 25% versus the bottom 25%. Analyses that were performed that did not generate statistically significant results are marked “NS” or “not significant”. An “X” designates an analysis that was not performed.

**Fig 3:**
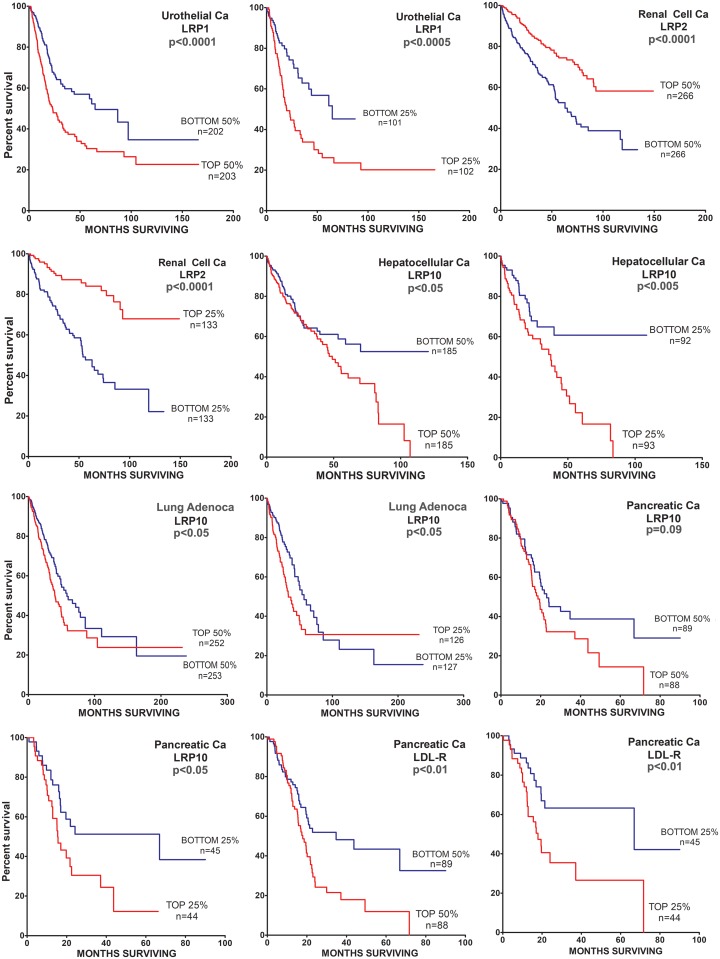
Patient survival curves that demonstrate significant effects of LRP mRNA expression on survival. Only highly expressed LRPs were analyzed for their effects on patient survival, as summarized in Table 1. For each gene and cancer, two curves are shown. The first stratifies the entire provisional dataset so that cases in which the gene of interest is expressed above the median value (top 50%) are compared with cases in which the gene of interest is expressed below the median value. In the second graph, populations in which the gene of interest is expressed in the top 25% or in the bottom 25% are compared. The identical datasets were mined to generate both plots. The cohort size or “n” is shown for each graph. The p-value is listed.

Although we examined the effects of *LRP1* mRNA expression on survival in all ten malignancies, *LRP1* mRNA expression demonstrated a significant correlation with patient survival only in urothelial carcinoma of the bladder. In this cancer, high levels of *LRP1* mRNA expression were associated with decreased survival in the 50%/50% comparison (p<0.0001) and in the 25%/25% comparison (p<0.0005) (Fig 3). The correlation between *LRP1* mRNA expression and patient survival in urothelial carcinoma was unanticipated because *LRP1* mRNA expression was relatively low in this cancer compared with other malignancies.

In renal cell carcinoma of the kidney, increased *LRP2* mRNA expression was associated with substantially improved patient survival. This is the only cancer in which we detected a correlation between high levels of expression of an LRP and increased patient survival. We speculated that LRP2 may represent a biomarker for more differentiated tumors.

We examined the effects of *LRP4* mRNA expression on patient survival in glioblastoma. We examined the effects of *LRP5* mRNA expression on patient survival in six cancers. These analyses failed to reveal significant correlations. Increased *LRP10* mRNA expression was associated with decreased patient survival in hepatocellular carcinoma, lung adenocarcinoma, and pancreatic adenocarcinoma. *LDLR* mRNA expression was associated with decreased patient survival in pancreatic adenocarcinoma.

## Discussion

Numerous studies have analyzed LRP expression and the function of LRPs in cancer; many of these studies have been performed using preclinical model systems. This paper does not review these previous studies comprehensively. Instead, we examined existing data regarding LRP gene family members in large provisional TCGA datasets and focused on mRNA abundance and possible correlations between LRP mRNA expression and cancer progression as determined by overall patient survival. We did not consider somatic or germline mutations in LRPs that may alter LRP structure and/or function. We also did not attempt to determine whether LRP expression varies with cancer properties such as grade. Patient populations are not stratified based on age, gender, or treatment protocols. These factors all may influence survival.

Examining glioblastoma in the context of gliomas in general provides an example of how the approach applied in database mining may influence the conclusions drawn. Glioblastomas are high-grade or grade-4 gliomas, which are differentiated from less aggressive low-grade (2/3) gliomas [22]. Ceccarelli et al. [23] recently published a dataset of RNA-Seq mRNA expression results comparing gliomas of different grades. In this dataset, the absolute abundance of *LRP1* mRNA was decreased in grade-4 gliomas or glioblastomas, compared with grade-2 and grade-3 gliomas. As a result, when the effects of LRP1 mRNA abundance on patient survival were examined, considering all gliomas comprehensively (grades 2+3+4), high levels of *LRP1* mRNA were significantly associated with improved patient survival. This correlation, however, most likely reflects *LRP1* functioning as a surrogate biomarker for low-grade gliomas. When survival of glioblastoma patients was examined in isolation, using the same dataset [23], *LRP1* mRNA expression failed to be significantly correlated with patient survival, although there was a trend in which increased *LRP1* mRNA was associated with decreased patient survival. The identical, non-significant trend was apparent in the provisional TCGA dataset. When we compared glioblastomas that expressed *LRP1* mRNA in the top 10% with tumors that expressed *LRP1* in the bottom 10%, increased *LRP1* expression was significantly correlated with decreased patient survival (p = 0.026, n = 106) [23]. These results and the high level of *LRP1* expression in human glioblastoma justify further studies to understand the function of this receptor in this malignancy.

Expression of *LRP2* in renal cell carcinoma has been reported previously [24]. Germ-line polymorphisms in the *LRP2* gene may be associated with increased risk for recurrence in prostate cancer [25] and somatic mutations in *LRP2* have been identified in gastric cancer [26]. Our analysis of patient survival data using TCGA datasets demonstrated a correlation between high levels of *LRP2* mRNA expression and increased patient survival in renal clear cell carcinoma. No correlation was observed in breast cancer. Andersen et al. [27] showed that in melanoma cells in culture, LRP2 promotes cell survival and proliferation. We did not observe a significant difference in patient survival when *LRP2* expression was examined in cutaneous melanomas in patients.

LRP5 and LRP6 are Wnt co-receptors that regulate β-catenin signaling [28–30]. Other members of this signaling pathway are well-characterized oncogenes and tumor suppressors, which when mutated in the germline or in cancer tissue, regulate carcinogenesis and cancer progression [31, 32]. Elegant studies have demonstrated regulation of β-catenin activity by LRP5 and LRP6 [33–37]. Because of its higher abundance, we chose to examine the effects of *LRP5* mRNA expression on patient survival in six different malignancies, including colorectal adenocarcinoma, and failed to demonstrate a significant effect. By contrast, high levels of *LRP10* mRNA expression correlated with decreased patient survival in hepatocellular carcinoma, lung adenocarcinoma, and pancreatic adenocarcinoma. *LRP10* mRNA has been identified in normal brain, muscle, heart, liver, kidney, and lungs [6, 38].

Very few mechanistic studies have addressed mechanisms by which LRP10 may regulate cancer progression. It is known that LRP10 traffics between the *trans*-Golgi network, endosomes, and the plasma membrane [39, 40]. LRP10 also has been identified as a negative modulator of Wnt/β-catenin signaling [41]. LRP10 interacts with Phosphatase of Regenerating Liver (PRL) gene family members, which are reported to regulate tumorigenesis and cancer metastasis [42]. The results of our TCGA analysis justify additional work to elucidate the function of LRP10 in cancer.

In patients with adenocarcinoma of the pancreas, high levels of *LDLR* mRNA expression were associated with worsened patient survival. This finding is particularly interesting because an important role for LDLR has been established previously in a transgenic mouse model of spontaneous pancreatic ductal adenocarcinoma [43, 44]. Apparently, in mouse pancreatic cancer, hypoxia is extensive and activation of lipid metabolism promotes tumor cell survival and cancer progression. The results presented here suggest that similar pathways may be operational in humans with pancreatic cancer, a malignancy for which current therapies are generally inadequate [45].

A unique characteristic of many LRPs, compared with most other receptors, is the ability to bind numerous ligands [4]. Because of this characteristic, LRPs may be targets for cancer therapeutic design even when expression does not correlate with patient survival. LRP1 provides an example. By recruiting distinct co-receptors in response to different ligands, LRP1 elicits ligand-specific cell-signaling responses [46–48]. If different LRP1 ligands are able to generate distinct signaling responses in cancer cells, it may be possible to design LRP1 ligands that drive tumor cell physiology in a manner that favors a cure.

## Materials and methods

### Mining The Cancer Genome Atlas (TCGA)

RNA-Seq V2 RSEM gene expression data were mined from TCGA, which is an open-access resource with datasets on a variety of malignancies. We examined provisional TCGA datasets using the cBioPortal for Cancer Genomics database [49, 50], which may be accessed at www.cbioportal.org. These datasets combine published data with data obtained from cancer samples examined subsequent to publication. The number of patients in each dataset at the time we mined TCGA is shown in Table 1. TCGA data were downloaded as text files. Bar graphs and vertical scatter plots were generated in Graphpad Prism 5.

### Identification of gene standards

Mean RNA-Seq V2 RSEM expression values and SDs were obtained for each gene in the ten provisional TCGA datasets, corresponding to the ten solid malignancies of interest. For each gene and solid tumor, a CV was calculated. The genes were then ranked based on the CV so that the gene with the lowest CV was number 1. We then averaged the CV rankings of each gene across the ten solid tumors and identified three genes with the lowest mean CV. These three genes are presented as standards to display the minimum anticipated variability in gene expression across different specimens of the same malignancy. To determine variability in gene expression across different malignancies, the mean expression levels in all ten solid tumors were averaged. The SDs and CVs were then determined.

### Patient survival analyses

For each malignancy, patient survival data are available in TCGA for only a fraction of the cases. This fraction is indicated in Fig 3. LRPs that were most abundantly expressed in each cancer were selected for patient survival analysis. Survival was compared for patients in which expression of the gene in the cancer of interest was in the top 50% versus the bottom 50%. A second analysis was performed comparing the top 25% and lowest 25%. Survival results were subjected to Mantel-Cox log-rank test using GraphPad Prism 5 (GraphPad Software). Differences in survival were considered statistically significant when the analysis achieved *p*<0.05.
